# ^125^I brachytherapy in the palliation of painful bone metastases from lung cancer after failure or rejection of conventional treatments

**DOI:** 10.18632/oncotarget.7584

**Published:** 2016-02-23

**Authors:** Zhanwang Xiang, Zhiqiang Mo, Guohong Li, Saba Gilani, Zhihui Zhong, Tao Zhang, Fujun Zhang, Fei Gao

**Affiliations:** ^1^ Sun Yat-sen University Cancer Center, State Key Laboratory of Oncology in South China, Collaborative Innovation Center for Cancer Medicine, Imaging and Intervention Therapy Department, Guangzhou 510060, Guangdong, China; ^2^ Midtown Medical Center, Columbus, GA 31901, USA

**Keywords:** pain, ^125^I seed, brachytherapy, bone metastases, lung cancer

## Abstract

**Purpose:**

This study sought to assess the safety and effect of ^125^I seed implantation for palliation of painful bone metastases from lung cancer after failure or rejection of conventional treatments.

**Materials and Methods:**

89 patients with painful bone metastases secondary to lung cancer were consented and enrolled in this study from June 2013 to May 2015. All patients had failed or refused conventional treatments underwent percutaneous CT-guided ^125^I seed implantation. The Brief Pain Inventory (BPI) was used to measure pain intensity prior to treatment (T_0_), 2, 4, 6, 8 and 12 weeks (T_2_, T_4_, T_6_, T_8_ and T_12_) after treatment in a 24-hour period. Analgesic, quality of life (QOL) scores and complications were also recorded. Four patients were excluded as they were lost to follow-up or had incomplete data.

**Results:**

85 patients with 126 bone metastases from lung cancer were treated. There were significantly lower scores after treatment in the visual analog scale (VAS) and analgesic. The VAS scores for worst pain was 6.3±1.8 at T_0_. At T_2_, T_4_, T_6_, T_8_ and T_12_, the score in a 24-hour period decreased to 4.9±1.2 (P<0.01), 3.7±1.3 (P<0.01), 3.4±1.2 (P<0.01), 2.6±0.9 (P<0.01), and 1.4±0.8 (P<0.01) respectively. Comparison of QOL scores showed improvements including sleep, appetite, spiritual state, and fatigue at T_2_, T_4_, T_6_, T_8_ and T_12_ when compared to T_0_. No serious complications or massive bleeding were observed.

**Conclusions:**

^125^I brachytherapy is a safe and effective method for palliation of painful bone metastases from lung cancer after failure or rejection of conventional treatments.

## INTRODUCTION

Bone was a common site of metastasis for patients with lung cancer, particularly non-small cell lung cancer, approximately 30-40% would appear skeletal metastases [1]. At the same time, pain was the most common clinical symptom of bone metastases and seriously decreased the QOL of tumor patients [2]. Unfortunately, more than half of lung cancer patients with painful bone metastases were difficult to obtain clinical remission during the treatment [3]. Thus, pain relief control has become an important part of the comprehensive treatment of lung cancer, and played an irreplaceable role in improving QOL.

Currently, conventional treatments were defined as bone metastases secondary to lung cancer had received local control therapies (external beam radiation therapy (EBRT), chemotherapy, analgesics and so on) before ^125^**I** brachytherapy [4]. Palliative therapy was seen as the standard of care for painful bone metastases from lung cancer, previous studies suggested that traditional therapies had achieved a certain effect [5, 6]. For focal metastases, chemotherapy and EBRT remained the most common methods, particularly EBRT was been reported to achieve remission in 60% of patients [7]. For diffuse metastases, treatment options include hormone therapy, radiopharmaceuticals, bisphosphonates, and analgesic agents.

Although a majority of traditional treatments have been used to treat painful bone metastases, the results can be disappointing [8]. Recent studies have shown that multiple minimally invasive, image-guided ablation techniques are emerging as safe, efficacious, and durable palliative therapies [9, 10]. Radiofrequency (RF) ablation has been the most studied thermal ablative technique demonstrating significantly reduced pain in patients who were not palliated by conventional therapies [11]. However, the thermal ablative techniques have some disadvantages including an increased possibility of pain and intra or post procedural injury to critical structures within the ablation zone [12].

Previous studies had shown that ^125^I seed implantation was an acceptable and useful minimally invasive therapy for primary lung tumor or metastatic lymph node resulting in a high local control rate making it an alternate approach for achieving remission [13, 14]. However, ^125^I brachytherapy for the palliation of painful bone metastases from lung cancer after failure or rejection of conventional treatments has not been reported. The purpose of this study is to report such data in patients with skeletal metastases secondary to lung cancer.

## RESULTS

### Patient and treatment characteristics

The patient, tumor, and brachytherapy characteristics were delineated in Table 1. Patients ranged in age from 25 to 82 years old, with 54 males and 31 females. The histological distribution rate of small cell carcinoma, adenocarcinoma, squamous carcinoma, large cell carcinoma, adenosquamous carcinoma was 18.8%(16/85), 43.5% (37/85), 22.4%(19/85), 8.2%(7/85), 4.7%(4/85), 2.4%(2/85), respectively. A single lesion was treated in 53 patients while two lesions were treated in 26 patients and three lesions treated in 7 patients, for a total of 126 tumor treated. A majority of patients received prior EBRT or chemotherapy to the treated site and had failed to achieve pain relief prior to enrollment into our study. Eleven patients (12.9%) had not received prior treatment before brachytherapy. The total number of implanted seeds was 2896, with an average of 23 ±6.5 per lesion.

**Table 1:** Characteristics of patient, tumor, and brachytherapy

Characteristics	Value
No. of patients (female/male)	31/54
age, y (±SD) Range	52 (±13) 25-82
Mean Karnofsky performance status (±SD)	70 (±2.7)
Previous treatment External beam radiation therapy Chemotherapy EBRT and chemotherapy No EBRT or chemotherapy	53/85 (62.4%) 45/85 (52.9%) 31/85 (36.5%) 11/85 (12.9%)
Opioid analgesics at presentation	69/85 (81.2%)
Primary lung tumor type histology (N=85) Small cell carcinoma Adenocarcinoma Squamous carcinoma Large cell carcinoma Adenosquamous carcinoma Others	16 (18.8%) 37 (43.5%) 19 (22.4%) 7 (8.2%) 4 (4.7%) 2 (2.4%)
Bone metastases location (N=126) Rib/chest wall Thoracic /Lumbar vertebra Iliac/ischium/pubic bones Sacrum Scapula Sternum Clavicle Acetabulum	55(43.7%) 26(20.6%) 23(18.2%) 13(10.3%) 4(3.2%) 3(2.4%) 1(0.8%) 1(0.8%)
Mean metastases diameter, cm (±SD) ≤2 >2, ≤4 >4, ≤6	3.1 (±0.8) 45/126(35.7%) 72/126(57.1%) 9/126(7.1%)
Metastases numbers 1 2 3	53/85(62.4%) 26/85(30.6%) 7/85(8.2%)
Type of bone metastases (N=85) Osteolytic Osteoplastic Mixed	48(56.5%) 25(29.4%) 12(11.5%)
Total number of ^125^I seed implantation Mean±SD	2896 23±6.5

Abbreviations: EBRT, external beam radiation therapy; SD, standard deviation.

### Responses of patient's pain tobrachytherapy

The effectiveness of pain palliation is summarized in Table 2. Analysis of pain control revealed significant difference among the worst and average pain scores in the various periods (p<0.01). The VAS scores for worst pain was 6.3±1.8 before brachytherapy. At T_2_, T_4_, T_6_, T_8_ and T_12_, the score in a 24-hour period decreased to 4.9±1.2, 3.7±1.3, 3.4±1.2, 2.6±0.9, and 1.4±0.8 respectively. Similarity, the score for average pain were 4.6±1.5, 3.5±1.2, 2.6±0.9, 2.1±0.7, 1.4±1.1 and 0.9±0.5 in a 24-hour period at T0, T2 (p<0.01), T4 (p<0.01), T6 (p<0.01), T8 (p<0.01) and T12 (p<0.01) respectively. The relief from pain was significantly improved from T_0_ to T_12_, whereas there was no significant difference in scores between T0 and T2 (P=0.27). The mean morphine equivalent 24-hour doses of 136.2±33.9mg at T0 and 109.4±29.8mg(p=0.57), 96.8±22.1mg(p=0.32), 108.4±27.2mg(p=0.64), 76.3±19.8mg(p=0.80), 39±6.8 mg(p=0.87) in the first 24 hours following T2, T4, T6, T8, T12 were not significantly different.

**Table 2:** Brief pain inventory-short form mean pain scores in a 24-hour period in each treatment period and distribution of pain severity scores

	T_0_ (N=85)	T_2_ (N=82)	T_4_ (N=75)	T_6_ (N=65)	T_8_ (N=57)	T_12_ (N=33)
Worst pain (0-10) Score ±SD P	6.3±1.8	4.9±1.2 <0.01	3.7±1.3 <0.01	3.4±1.2 <0.01	2.6±0.9 <0.01	1.4±0.8 <0.01
Average pain (0-10) Score ±SD P	4.6±1.5	3.5±1.2 <0.01	2.6±0.9 <0.01	2.1±0.7 <0.01	1.4±1.1 <0.01	0.9±0.5 <0.01
Pain relief (0-100) Score ±SD P	54.4±18.1	61.2±15.8 0.27	65.8±12.1 <0.01	80.1±9.8 <0.01	88.3±6.9 <0.01	94.4±5.8 <0.01
Morphine-equivalent 24-hour dose (mg) Dose ±SD P	136.2±33.9	109.4±29.8 0.57	96.8±22.1 0.32	108.4±27.2 0.64	76.3±19.8 0.80	39.1±6.8 0.87
Mild	0(0)	11(13.4%)	19(25.3%)	24(36.9%)	32(56.1%)	22(66.7%)
Moderate	51(60%)	45(54.8%)	39(52%)	28(43.1%)	19(33.3%)	9(27.3%)
Severe	34(40%)	26(31.7%)	17(22.7%)	13(20%)	6(10.5%)	2(6.1%)
P		0.32	<0.01	<0.01	<0.01	<0.01

Abbreviations: SD, standard deviation.

Further comparison of the percentage distribution of patient pain severity showed the percentages of mild and moderate pain were significantly higher in posttreatment than prior treatment. the percentage of patients with mild and moderate pain was significantly difference at T_4_(p<0.01), T_6_(p<0.01), T_8_(p<0.01), T_12_(p<0.01) than T0, whereas there was no significant difference in scores between T_0_ and T_2_ (P=0.32) (Table 2).

### Differences in of quality-of-life scores

Comparison of QOL scores showed quality of sleep, appetite, spiritual state, fatigue and KPS at T_2_, T_4_, T_6_, T_8_ and T_12_ were all significantly better than T0 (Table 3). Sleep quality scores were 1.3±0.4, 1.9±1.2 (p<0.01), 2.5±1.4 (p<0.01), 2.9±1.2 (p<0.01), 3.7±0.9 (p<0.01) and 4.3±0.5 (p<0.01) in each period respectively indicating a statistically significant difference, although there was no significant difference in scores between T_0_ and T_2_ in the appetite(p=0.35), spiritual state(p=0.33), and fatigue(p=0.28) categories (Table 3).

**Table 3:** Differences of QOL scores in various periods

QOL	T_0_ (N=85)	T_2_ (N=82)	T_4_ (N=75)	T_6_ (N=65)	T_8_ (N=57)	T_12_ (N=33)
Sleep Score ±SD P	1.3±0.4	1.9±1.2 <0.01	2.5±1.4 <0.01	2.9±1.2 <0.01	3.7±0.9 <0.01	4.3±0.5 <0.01
Appetite Score ±SD P	2.0±0.8	2.6±0.9 0.35	3.1±1.2 <0.01	3.7±0.8 <0.01	3.8±0.6 <0.01	4.0±0.8 <0.01
Spiritual state Score ±SD P	1.9±0.6	2.3±1.1 0.33	3.7±0.9 <0.01	4.1±0.7 <0.01	4.7±0.2 <0.01	4.6±0.2 <0.01
Fatigue Score ±SD P	1.8±0.6	2.5±0.9 0.28	3.6±1.1 <0.01	3.8±0.9 <0.01	4.1±0.6 <0.01	4.4±0.5 <0.01
Mean KPS ±SD	70 ±2.7	75.8±3.4	82.5±3.8	84.6±4.6	88.2±2.6	92.5±2.8
P		0.46	0.54	0.69	0.32	0.87

Abbreviations: QOL, Quality-of-Life ; KPS, Karnofsky Performance Status ; SD, standard deviation.

### Local tumor control and complications

All local bone tumors after^125^I brachytherapy wrer stable according to Response Evaluation Criteria In Solid Tumors (RECIST) at 2 weeks on CT or PET-CT image and the range of tumor necrosis was gradually increasing with the follow-up prolonged. In addition, patients treated with ^125^I brachytherapy did not experience increased pain.

Several complications related to the procedure occurred during or after brachytherapy (Table 4). Small volume subcutaneous hemorrhage occurred in 17 patients(20%) related to applicator insertion along the course of subcutaneous vessels. Six patients(7.1%) presented local skin reaction after the procedure and recovered after expectant management. Follow up imaging revealed two patients(2.4%) had minor displacement of radioactive seeds and did not cause adjacent tissues harm. Severe complications such as massive bleeding and radiation pneumonitis were not seen in the entire study group.

**Table 4:** Complications of 125I brachytherapy

Complications	N (%)
A small amount of subcutaneous hemorrhage	20(17/85)
Local skin reaction	7.1(6/85)
Minor displacement of radioactive seeds	2.4(2/85)
Fever	1.2(1/85)
Granulocytopenia	1.2(1/85)
Pathologic fracture	0
Hydropneumothorax	0
Radiation pneumonia	0
Massive bleeding	0

## DISCUSSION

Up to 30 % of patients with lung cancer have bone metastases, and half of these develop intractable pain [15]. The etiology of bone cancer pain is multifactorial, including factors such as osteoclast-mediated bone remodeling, distortion of mechano-sensitive fibers following normal mechanical stress because of loss of tensile strength in the bone due to cancer, and production of nerve-stimulating tumor-derived products (cytokines, vasoactive peptides, growth factors) [16, 17].

Conventional therapies for painful bone metastases included systemic and local interventions. Chemotherapy and analgesic were systemically administered in select patients for palliation, but the effectiveness was variable [18, 19, 20]. The most common locoregional treatments was EBRT. A trial conducted by the Radiation Therapy Oncology Group (RTOG) including 1016 patients found complete pain relief in 53% of patients and partial relief in 83% with a mean duration of response of 12 and 20 weeks in these 2 groups, respectively [21]. Chow E et al. also reported a recent meta-analysis reviewing of 25 randomized controlled trials, showing similar duration of palliation response for the single and multiple fraction treatments for palliation of painful bone metastases, with median duration of response ranging from 12 to 28 weeks [22]. Although EBRT had really achieved success, some limitations were also found. First, most of lung cancer patients had received adjuvant radiation therapy for primary tumors, thereby limiting the potential for further EBRT to bone metastases. Second, it could be difficult for normal tissues to tolerate repeating radiation dose. Moreover, external-beam radiation cannot reach the dose for killing the bone tumor because of nearby vital organs.

Previously, multiple minimally invasive image-guided ablation strategies have been studied as treatments for painful bone metastases [23, 24]. Cryoablation and RF ablation have been the most studied techniques demonstrating significantly reduced pain in patients who are unresponsive or incompletely responsive to standard conventional therapies. Matthew R et al. reported that scores for worst pain using the BPI were 7.1 before treatment, 5.1, 4.0, 3.6, and 1.4 at 1,4, 8, and 24 weeks respectively [25]. Goetz et al. reported scores for worst pain were 7.9 prior to treatment, and reduced to 5.8, 4.5, 3.0, and 1.4, respectively at 1, 4, 8, and 24 weeks following RF ablation treatment [26]. In contrast to Cryoablation or RF ablation, patients in our study treated with ^125^I brachytherapy did not experience increased pain. A second advantage is providing complete irradiation to the local tumor, while it provided a lower dose to normal adjacent tissue.

In this study, we demonstrated that ^125^I brachytherapy was an effective, accurate method for palliation of painful bone metastases. We found that the scores for worst pain were 6.3±1.8 before brachytherapy, and 4.9±1.2, 3.7±1.3, 3.4±1.2, 2.6±0.9, and 1.4±0.8 at 2, 4, 6, 8 and 12 weeks respectively, the symptom of pain was decreased obviously from T_2_ to T_12_. These corresponding results further illustrated that ^125^I brachytherapy for palliation of painful bone metastases painful bone metastases was significant and feasible.

Another significant finding in this study was that ^125^I brachytherapy was effective at improving QOL. Comparison of QOL scores, the quality of sleep, appetite, spiritual state, fatigue and KPS at T_2_, T_4_, T_6_, T_8_ and T_12_ were all significantly better than at T_0_. We found that the treatment of pain was valuable, because the bone metastases were the only evidence of recurrence since lung cancer was controlled. Particularly encouraging were outcomes in five patients who were alive without pain or recurrence after^125^I brachytherapy. For these patients, ^125^I brachytherapy had been a curative treatment thus far.

The success of^125^I brachytherapy for pain palliation depended on the accurate placement of radioactive seeds within a known volume of tumor [27]. American Brachytherapy Society's “dual 90” guideline was that a cancer cure required that 90 % of the tumor volume got 90 % prescription dose [28]. Because radiation from^125^I seed dropped off with distance, our study showed that TPS can help peripheral tumor doses reach the MPD of 100-140 Gy and ensure that more than 95% of the tumor get 100% prescription dose [29]. Thus, the tumor target received adequate dose without increasing radiation to surrounding normal tissue. Using CT guidance, an depiction of the tumor volume could be seen and all patients with bone metastases from lung cancer were treated successfully, A small amount of subcutaneous hemorrhage occurred in 17 patients(20%), probably because of injury of subcutaneous vessels. Six patients(7.1%) presented local skin reaction after the procedure and recovered after expectant treatment. No massive bleeding or serious complication were observed. These further illustrated that^125^I brachytherapy was safe and effective.

The main limitations of this study were small sample size and a short duration of follow up. Also, a control group consisting of patients treated with EBRT or chemotherapy should be needed, further comparison and longer follow-up were desired for future analysis. Although pathology sub-types of lung cancer metastases with the 85 patients were clarified, it is not clear which pathology sub-type of metastasis is more sensitive to ^125^I brachytherapy. Thus, the next research is to explore the responses and sensibility about different pathology sub-types of lung cancer metastases for ^125^I brachytherapy. Last, responses to the pain score and QOL rating systems were provided by patients who were not previously familiar with these surveys introducing subjectivity into their assessments.

In conclusion, CT-guided ^125^I seed implantation is a safe, effective, and accurate treatment for patients with painful bone metastases from lung cancer, who had failed or refused conventional therapies. Both QOL and pain palliation are achieved by this locoregional approach to skeletal metastases. Studies, in larger cohorts, can help substantiate this claim and may help broaden application of this therapy to primary bone tumors.

## MATERIALS AND METHODS

### Patients

The study was approved by the ethics committees at Sun Yat-sen University Cancer Center. Between June 2013 and May 2015, a total of 89 lung cancer patients with painful bone metastases (a score of ≥4 worst pain on a scale of 0 to 10 over the past 24 hours according to BPI-Short Form) were recruited and consented for participation in this study. Most patients had previously pain management to bone metastases with external beam radiation therapy(EBRT), chemotherapy, analgesics, or patients had refused therapies, but the remission of pain was unsatisfied. These patients underwent CT-guided ^125^I brachytherapy After excluding patients lost to follow-up or for whom data was missing (n=4), data for the remaining 85 patients with 126 treated metastases was analyzed.

All enrolled patients met the following criteria: (a) Bone metastases from lung cancer were histologically or cytologically proven with imaging concordance; (b) Score of ≥4 worst pain over the past 24 hours according to BPI-Short Form; (c) Failure or refusal of conventional treatments (radiation therapy, chemotherapy, and/or analgesic medicines); (d) The number of bone lesions ≤3 with the size of each lesion ≤6cm; (e) Expected survival time ≥3 months; (f) Karnofsky performance status of ≥70; (g) Absence of coagulopathy (prothrombin activity >40 or platelet count >50,000/μL); (h) Ability to tolerate ^125^I brachytherapy with CT-imaging; (i) Absence of impending fracture; (j) No severe cardiopulmonary dysfunction.

### ^125^I seed

The type of each implanted ^125^I seed (Yunke Pharmaceutical Limited Liability Company, Chengdu, China) was 6711–1985, with a diameter of 0.8 mm, and a length of 4.5 mm. The central source of the particles was a ^125^I radionuclide silver rod, with a diameter of 0.5 mm, and a length of 3.0 mm. The matched peripheral dose was 110–140 Gy, and the average energy was 27–32 KeV. Each deposit had an initial activity of 0.8 mCi and a half-life of 59.6 days, release of continuous low-dose γ-ray and soft X-ray (5% of 35 keV and 95% of 28 keV, respectively) after decaying into the organization. The ^125^I seeds had effective anti-tumor activity at a radius of 1.7 cm. Within 8–10 months, 93–97% of the brachytherapy dose was delivered.

### ^125^I brachytherapy

**Figure 1:**
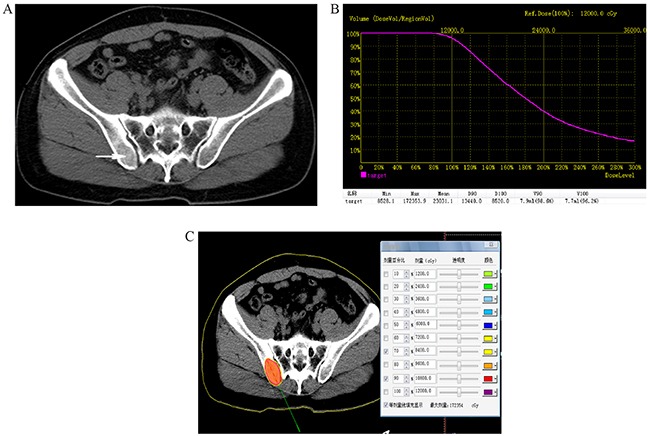
**A.** Preoperative computed tomography (CT) image was obtained for targeting bone areas of interest, which showed one lesion in right iliac bone (arrowhead). **B.** Dose volume histograms (DVH). The prescription dose is 120 Gy during the planning. A total of 90% of the tumor target (D90) received 134.4 Gy, and 96.2% of the tumor received 100% of the prescribed dose (V100 = 96.2%). **C.** Isodose curves plotted by the treatment planning system (TPS), the planning target volume (PTV) edge was covered by isodose curve from 70% to 90%.

Before ^125^I brachytherapy, 5mm axial enhanced CT images were obtained in all patients (Figure 1A). Treatment was mapped and dosed for each patient using a computerized treatment planning system (TPS) (RT-RSI, Beijing Atom and High Technique Industries Inc, Beijing, China).

A careful delineation of the gross tumor volume (GTV), planned target volume (PTV) and surrounding vital organs (e.g. spinal cord) was sought in every CT slice. PTV is defined as a 1.5 cm of expansion external to the GTV. The prescribed dose was averaged 120 Gy (range 100-140 Gy). Based on three orthogonal diameters within the target tumor and a prescribed matched peripheral dose (MPD) of averaging 120 Gy, TPS generated a dose-volume histogram (DVH), isodose curves of different percentages, and calculated the position of brachytherapy applicator, dose and number of implanted seeds (Figure 1B, 1C). The PTV edge was accounted by the 70%-90% isodose curve. The entry site and path of the needle were determined to avoid vital organs and tissues.

**Figure 2:**
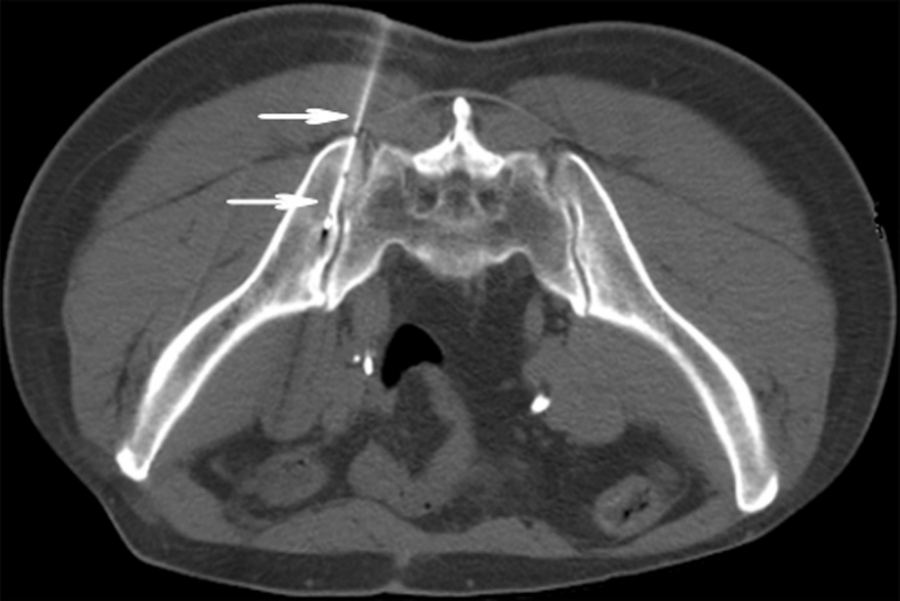
CT -guided percutaneous ^125^I seed implantation was performed according to preoperative TPS plan, a 18 G spinal needle reached the tumor (arrowhead). From deep to shallow, the particles were released.

**Figure 3:**
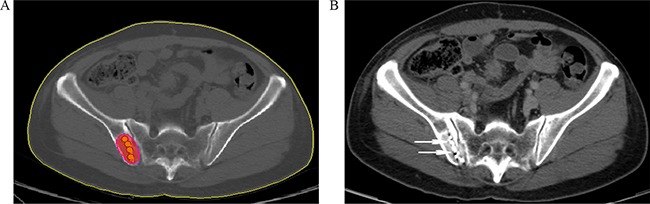
**A.** Verification of post-^125^I brachytherapy. the PTV edge was covered by isodose curve. **B.** Two weeks after brachytherapy, the tumor kept stable, with well distributed radioactive seeds remaining (arrowhead).

On the day of the procedure, the patient was pasitioned on the CT gantry and treatment site was localized. 5mm axial slices were obtained to delineate the upper and lower borders of the tumor. After local infiltration anesthesia with 5-15 mL of 1% lidocaine (Liduokayin; Yimin, Yichang, China), an 18 G spinal needle (Yunke Pharmaceutical Limited Liability Company, Chengdu, China) reached the farthest tumor edge, but was kept at approximately or less than 5 mm of the border (Figure 2). A turntable or clip implant gun (Yunke Pharmaceutical Limited Liability Company, Chengdu, China) was then attached to the applicator for implantation. From deep to shallow, the particles were released while retracting the needle and keeping adjacent particles at a distance of 5–15 mm. To avoid introprocedural complications, all of the spinal needles were retained until the implantation was completed and then removed simultaneously. A completion CT scan was performed to assess for postoperative complications such as bleeding. The last scan image was also reviewed to verify the position and intensity of ^125^I seeds according to TPS (Figure 3A, 3B). If a lesion demonstrated insufficient radioactivity, the procedure was repeated for additional ^125^I implantation.

### Follow up

#### Efficacy assessment

A dynamic enhanced CT and clinical hematological examination (WBC, RBC, platelet counts, s-hemoglobin, and coagulation profile) were performed every two weeks for the first two months, then once every month after 2 months. Each patient was asked to answer relative questions with respect to the lesion that was to be treated by interview or telephone. we assessed pain intensity using the BPI-Short Form (a validated visual analog scale with scores ranging from 0-10) and personal analgesic use in a 24-hour period at T_0_, T_2_, T_4_, T_6_, T_8_ and T_12_, respectively. The worst pain, least pain, and average pain scores from 0 to 10 (0, no pain; 10, maximum pain intensity) were recorded. Relief of pain through the use of pain treatments or medications were scored on a 0–100 % scale in 24 hours (0 %, no relief; 100 %, complete relief). To further evaluate the results, pain scores of 0–3 were categorized as mild; 4–6, as moderate; and 7–10, as severe pain. The percentage distribution of patients whose worst pain score decreased in each category was compared during various periods.

Quality-of-life factors including sleep, appetite, spiritual state, and fatigue were rated by patients at T_0_, T_2_, T_4_, T_6_, T_8_ and T_12_ by using a five-point categoric scale (1 = worst, 2 = bad, 3 = mild, 4 = normal, 5 = very good), similarly, KPS also was recorded. The changes in scores during these intervals, indicating recovery of QOL, were calculated and analyzed.

#### Safety assessment

Those patients who underwent percutaneous ^125^I seed implantation underwent cardiac monitoring. All postoperative symptoms and complatations were recorded in detail, including a small amount of subcutaneous hemorrhage, local skin reaction, minor displacement of radioactive seeds, fever, granulocytopenia, pathologic fracture, hydropneumothorax, radiation pneumonia and massive bleeding. We calculated the percentage of various complications in order to further evaluated the safety of ^125^I seed implantation.

### Statistical analysis

The statistical software package SPSS version 10.0 was used for statistical analyses. Brachytherapy effectiveness was assessed by comparing the mean pain scores and the percentage distribution of patients reporting scores in each pain category (mild, moderate, and severe) for each time period. The mean pain scores differences among various treatment periods were analyzed by paired t tests supplemented with repeated-measures analysis of variance (ANOVA), comparing them with scores at T_0_. Scores for QOL factors were compared using the same methods. The percentage distribution of patients in each pain category during each interal was analyzed with the Cochran-Mantel- Haenszel x^2^ test. A P value of less than 0.05 was considered an indicator of a statistically significant difference.
